# Higher Trimethylamine-*N*-Oxide Plasma Levels with Increasing Age Are Mediated by Diet and Trimethylamine-Forming Bacteria

**DOI:** 10.1128/mSystems.00945-21

**Published:** 2021-09-14

**Authors:** Silke Rath, Katharina Rox, Sven Kleine Bardenhorst, Ulf Schminke, Marcus Dörr, Julia Mayerle, Fabian Frost, Markus M. Lerch, André Karch, Mark Brönstrup, Dietmar H. Pieper, Marius Vital

**Affiliations:** a Microbial Interactions and Processes Research Group, Helmholtz Centre for Infection Research, Braunschweig, Germany; b Department of Chemical Biology, Helmholtz Centre for Infection Research, Braunschweig, Germany; c German Center for Infection Research (DZIF), Hannover-Braunschweig Site, Helmholtz Center for Infection Research, Braunschweig, Germany; d Institute of Epidemiology and Social Medicine, University of Münster, Münster, Germany; e Department of Neurology, University Medicine Greifswald, Greifswald, Germany; f Department of Internal Medicine B, University Medicine Greifswald, Greifswald, Germany; g German Centre for Cardiovascular Research (DZHK) partner site Greifswald, Greifswald, Germany; h Department of Medicine II, University Hospital Munich, Ludwig-Maximillian University Munich, Munich, Germany; i Department of Medicine A, University Medicine Greifswald, Greifswald, Germany; j Institute for Medical Microbiology and Hospital Epidemiology, Hannover Medical School, Hannover, Germany; University of Pennsylvania

**Keywords:** aging, cardiovascular disease, diet, microbiota, trimethylamine

## Abstract

The gut microbiota-dependent metabolite trimethylamine-*N*-oxide (TMAO) is linked to an increased risk for cardiovascular diseases. Trimethylamine (TMA), which is subsequently oxidized to TMAO in the liver, is formed by intestinal bacteria via distinct biochemical routes from dietary precursors that are enriched in animal product-based foods. To get a full picture of the entire process of the diet > gut microbiota > TMAO axis, we quantified potential TMA-forming gut bacteria and plasma metabolites using gene-targeted assays and targeted metabolomics on a subsample (*n* = 425) of a German population-based cohort study. We specifically compared persons reporting daily meat intake with those that rarely or never consume meat. While meat intake did not predict TMAO plasma levels in our study, two major bacterial TMA-forming pathways were linked to the metabolite’s concentration. Furthermore, advancing age was strongly associated with TMAO. Construction of a structural equation model allowed us to disentangle the different routes that promote higher TMAO levels with increasing age, demonstrating, for the first time, a functional role of gut microbiota in the process, where specific food items augmented abundances of TMA-forming bacteria that were associated with higher TMAO plasma concentrations. Analyses stratified by age showed an association between carotid intima-media thickness and TMAO only in individuals >65 of age, indicating that this group is particularly affected by the metabolite.

**IMPORTANCE** Many cohort studies have investigated the link between diet and plasma TMAO levels, reporting incongruent results, while gut microbiota were only recently included into analyses. In these studies, taxonomic data were recorded that are not a good proxy for TMA formation, as specific members of various taxa exhibit genes catalyzing this reaction, demanding function-based technologies for accurate quantification of TMA-synthesizing bacteria. Using this approach, we demonstrated that abundances of the main components leading to TMAO formation, i.e., TMA precursors and TMA-forming bacteria, are uncoupled and not governed by the same (dietary) factors. Results emphasize that all levels leading to TMA(O) formation should be considered for accurate risk assessment, rejecting the simple view that diets rich in TMA precursors directly lead to increased plasma levels of this hazardous compound. The results can assist in developing strategies to reduce TMAO levels, specifically in the elderly, who are prone to TMAO-associated diseases.

## INTRODUCTION

The gut microbiota-dependent metabolite trimethylamine-*N*-oxide (TMAO) has been linked to an increased risk for developing cardiovascular and renal diseases independently of traditional risk factors in several studies (1–3). Moreover, it has been suggested that TMAO also plays a role in obesity and type 2 diabetes (4). Mechanistically, TMAO was shown to act through promoting inflammatory mechanisms and enhancing platelet reactivity (5, 6). The formation of TMAO is a complex process involving specific gut microbes that metabolize the dietary-derived precursors choline, betaine, and carnitine to trimethylamine (TMA), which subsequently crosses the gut epithelial barrier to enter the hepatic portal vein, where it is transported to the liver and oxidized to TMAO by flavin monooxygenases (FMOs) (5).

TMA is formed by bacteria via distinct biochemical routes from choline, carnitine, and betaine, with choline TMA lyase (CutC), carnitine oxygenase (CntA), and betaine reductase (GrdH) as the key enzymes of respective pathways (7, 8). While diverse diets contain carnitine and choline, those compounds are specifically enriched in animal product-derived foods such as red meat, eggs, and shellfish (9), whereas betaine is mostly found in plants (10). Bacteria are in competition with the host, who absorbs TMA precursors as essential nutrients, and formation of TMA(O) is, hence, governed by a fragile balance between diet, gut bacteria, and host physiology (11). Several interventional and population-based cohort studies have investigated the link between diet and plasma TMAO levels. Whereas the majority of studies reported a positive correlation between meat intake and TMAO concentrations (12, 13) and reduced TMAO levels on vegetarian diets (14), respectively, several studies did not find such correlations (15, 16) or described increased TMAO concentrations with specific plant-based foods (17, 18). Recently, the missing link between diet and TMAO concentration, namely, gut microbiota, was included in analyses, where specific bacterial taxa have been assessed for their association with TMAO formation (19, 20). However, taxonomic information for gut microbiota does not serve as a good predictor for TMA formation, as various taxonomic groups, with typically only a subset of members of a given taxon, encode enzymes catalyzing this reaction and function-based technologies are, hence, required for accurate quantification of TMA-forming bacteria (21, 22). Little is known about the ecophysiology of those bacteria, and although it is tempting to consider TMA precursors as major selection factors, other substances such as plant-derived polysaccharides are most probably more central for their growth, potentially decoupling precursor-rich diets from nutritional conditions that promote growth of TMA-forming bacteria (8). Furthermore, certain host features, particularly advancing age and (male) sex, were associated with increased TMAO levels (17, 23); however, it is currently unclear which specific factors drive those increases.

To get a comprehensive picture of the entire diet > gut microbiota > TMAO axis, we quantified potential TMA-forming bacteria and plasma metabolites using gene-targeted assays and targeted metabolomics on samples from a German population-based cohort study. We specifically compared subjects who reported frequent meat consumption with those that rarely or never consumed meat in the general population.

## RESULTS

We investigated the associations of all major components of the diet > gut microbiota > TMA(O) axis, including data on general dietary habits and quantification of plasma metabolites as well as potential TMA-forming bacteria from 425 individuals of the SHIP cohort in northeast Germany. Samples (*n* = 425) were drawn from participants of two distinct dietary groups, i.e., persons who reported daily meat consumption versus those primarily fostering a vegetarian diet. Their main characteristics, along with plasma concentrations of the TMA precursors betaine, carnitine, and choline as well as TMAO and abundances of genes encoding betaine reductase (*grdH*), carnitine oxygenase (*cntA*), and choline TMA lyase (*cutC*) from main TMA-forming pathways of gut microbiota, are summarized in Table 1.

**TABLE 1:** Differences in TMAO, BMI, plasma metabolite concentrations, and abundances of genes (*grdH*, *cntA*, and *cutC*) encoding key enzymes of main TMA-forming pathways in stool samples between the two dietary groups (rare meat consumption and daily meat consumption)^*a*^

Variable	Value			p.val	p.adj
	Overall (*n* = 425)	Rare meat consumption (*n* = 214)	Daily meat consumption (*n* = 211)		
Sex [no. (%)]				**<0.01**	
Female	211 (49.6)	153 (71.5)	58 (27.5)		
Male	214 (50.4)	61 (28.5)	153 (72.5)		
Age (yrs)				**<0.01**	
Mean (SD)	52.6 (15.6)	55.1 (15.4)	50.1 (15.6)		
Median (min, max)	53.0 (22.0, 88.0)	56.0 (23.0, 88.0)	51.0 (22.0, 81.0)		
BMI (kg/m^2^)				**0.021**	**<0.01**
Mean (SD)	28.2 (5.33)	27.6 (5.05)	28.8 (5.55)		
Median (min, max)	27.6 (18.3, 52.9)	26.6 (18.3, 44.5)	28.4 (18.3, 52.9)		
Betaine (μM)				**0.018**	**0.003**
Mean (SD)	19.9 (13.7)	18.8 (13.7)	20.9 (13.7)		
Median (min, max)	16.2 (1.48, 120)	15.2 (1.48, 78.2)	17.6 (4.73, 12)		
Carnitine (μM)				**0.018**	**0.006**
Mean (SD)	46.5 (21.0)	44.6 (21.0)	48.6 (20.9)		
Median (min, max)	42.8 (4.99, 125)	41.0 (4.99, 124)	44.8 (14.1, 125)		
Choline (μM)				**0.039**	**0.018**
Mean (SD)	8.26 (3.75)	7.95 (3.87)	8.57 (3.61)		
Median (min, max)	7.51 (0.288, 27.7)	7.23 (0.288, 27.7)	7.80 (0.518, 22.8)		
*grdH* (×10^5^)^*b*^				0.879	0.812
Mean (SD)	0.165 (0.295)	0.167 (0.315)	0.163 (0.274)		
Median (min, max)	0.073 (0.007, 3.24)	0.0723 (0.007, 3.24)	0.0751 (0.007, 1.95)		
*cntA* (×10^5^)^*b*^				**0.014**	0.062
Mean (SD)	1.59 (8.32)	2.37 (11.3)	0.808 (2.84)		
Median (min, max)	0.029 (0.029, 134)	0.029 (0.029, 134)	0.029 (0.029, 23.4)		
*cutC* (×10^5^)^*b*^				**0.036**	0.119
Mean (SD)	1.43 (1.50)	1.54 (1.70)	1.32 (1.25)		
Median (min, max)	1.12 (0.042, 15.5)	1.19 (0.051, 15.5)	1.09 (0.042, 10.5)		
TMAO (μM)				0.746	0.759
Mean (SD)	3.27 (5.31)	3.34 (5.05)	3.20 (5.58)		
Median (min, max)	2.10 (0.373, 74.6)	2.14 (0.466, 57.4)	2.01 (0.373, 74.6)		

a *P* values of <0.05 are shown in boldface. Raw *P* values (p.val) based on linear regression analyses are given. Age was determined as an important explanatory variable (see the text), and *P* values adjusted for age (p.adj) are given as well. For sex, the χ^2^ test was used and no p.adj is available. min, minimum; max, maximum.

b Gene copies per 10 ng of DNA are given.

**FIG 1:**
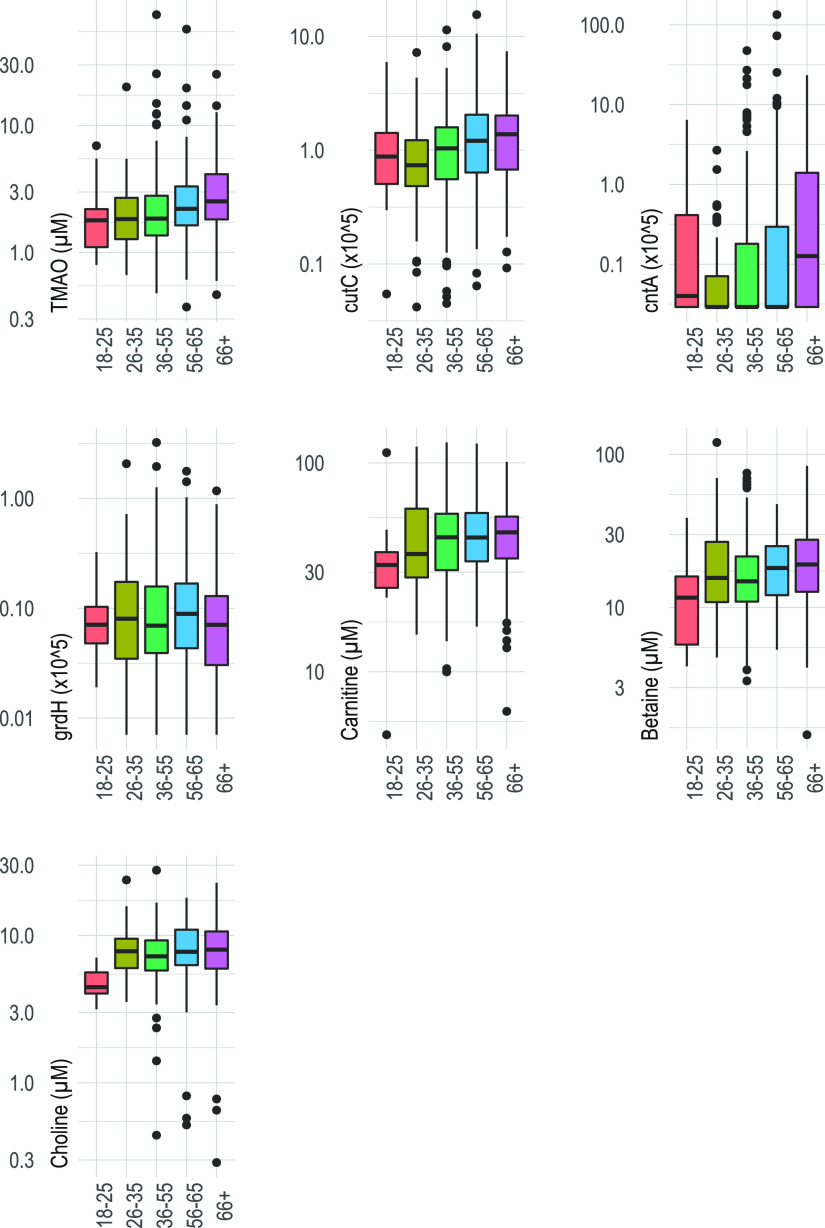
Plasma metabolite concentrations and abundances of genes (*grdH*, *cntA*, and *cutC*) encoding key enzymes of main TMA-forming pathways by age groups.

Overall, TMAO showed a mean concentration of 3.27 μM and a high variation between samples, with a standard deviation (SD) of ±5.31 μM observed (Table 1). Out of the precursors, carnitine showed the highest concentration, followed by betaine and choline. The *grdH* and *cutC* genes were detected in all 425 fecal samples, whereas *cntA* was found in only 42% of all samples. The relative abundances of *cntA*- and *cutC*-harboring bacteria were higher than for *grdH*. All three TMA precursor concentrations were significantly higher (*P* < 0.05) in individuals that reported daily meat consumption, whereas gene abundances of *cutC* and *cntA* displayed opposite patterns and were lower in abundance (*P* < 0.05) in that group; *grdH* abundance was not associated with any dietary group (Table 1). Higher meat consumption did not affect TMAO plasma levels (*P* = 0.75) in our study. Increasing age, however, was associated with all variables, namely, TMA-forming bacteria (*P* < 0.01, only *cutC* and *cntA*), precursor concentrations (*P* < 0.05, only carnitine and betaine), and TMAO levels (*P* < 0.01); descriptive visualizations of these associations based on stratified age groups are shown in Fig. 1 (summary statistics as well as the overall effect of age on those variables can be found in the supplemental material [Table S1]). While sex was not associated with TMAO levels (*P* = 0.60), concentrations of both TMA precursors (all three higher in males; *P* < 0.01) and TMA-forming bacteria (*cutC* higher in females; *P* < 0.05) were different between the two groups, even if adjusted for diet (Table S2). Sex was, however, not associated with age (males, 52.73 ± 16.28 years, females, 52.52 ± 15.03 years; *P* = 0.89). An observed positive association between body mass index (BMI) and TMAO concentrations (*P* = 0.032) was not sustained when adjusted for age (*P* = 0.274) (Table S3). BMI was not associated with precursors when adjusted for age or with the major TMA-forming bacteria carrying *cutC* and *cntA*, while a positive correlation with *grdH* was found (Table S3).

10.1128/mSystems.00945-21.2 TABLE S1 Differences in plasma metabolite concentrations and abundances of genes (*grdH*, *cntA*, and *cutC*) encoding key enzymes of main TMA-forming pathways in stool samples of subjects by age groups. Significance (p.age) was assessed based on continuous age data. Download Table S1, PDF file, 0.1 MB. Copyright © 2021 Rath et al. 2021 Rath et al. https://creativecommons.org/licenses/by/4.0/ This content is distributed under the terms of the Creative Commons Attribution 4.0 International license.

10.1128/mSystems.00945-21.3 TABLE S2 Effect of sex on TMAO, TMA precursors, and TMA-producing bacteria. Diet and age were determined as important explanatory variables, and *P* values adjusted for either of these parameters (p.adj.diet and p.adj.age) are given as well. Download Table S2, PDF file, 0.1 MB. Copyright © 2021 Rath et al. 2021 Rath et al. https://creativecommons.org/licenses/by/4.0/ This content is distributed under the terms of the Creative Commons Attribution 4.0 International license.

10.1128/mSystems.00945-21.4 TABLE S3 Effect of BMI on TMAO, TMA precursors, and TMA-producing bacteria. Age was determined as an important explanatory variable, and *P* values adjusted for this parameter (p.adj.age) are given as well. Download Table S3, PDF file, 0.1 MB. Copyright © 2021 Rath et al. 2021 Rath et al. https://creativecommons.org/licenses/by/4.0/ This content is distributed under the terms of the Creative Commons Attribution 4.0 International license.

Overall, TMAO concentrations were associated significantly (*P* < 0.05) with gene abundances of *cntA* and *cutC* (Table 2), indicating that levels of TMA-forming bacteria affect plasma levels of this harmful metabolite. Again, age had a profound effect on those associations. Next, we looked at the effects of TMA precursors and individual food items on TMA-forming bacteria in order to investigate how diet affects their abundances. Whereas the concentration of none of the TMA precursors was associated with any gene abundances (*P* > 0.05), the consumption of specific foods, namely, salad (*cntA*), fruits (*cutC*), and vegetables (*cutC*), had a positive effect on gene abundances (Table 2; detailed results for all dietary items recorded in the cohort are shown in Table S4). Furthermore, eggs and choline had a positive effect on TMAO levels. Overall, diet changed with age, where in particular increased consumption of fruits and a decreased proportion of daily meat eaters were observed (Table S5).

**TABLE 2:** Associations between abundances of genes (*grdH*, *cntA*, and *cutC*) encoding key enzymes of main TMA-forming pathways and TMAO plasma concentrations as well as dietary items^*a*^

Outcome	Predictor	p.val	p.adj.age	p.adj.sex
TMAO	*grdH*	0.868	0.949	0.858
TMAO	*cntA*	**0.044**	0.206	**0.037**
TMAO	*cutC*	**0.014**	0.069	**0.011**
*cntA*	Salad	**0.014**	**<0.01**	**0.039**
*cutC*	Fruits	**<0.01**	**0.013**	**0.011**
*cutC*	Vegetables	**0.038**	**0.047**	0.077

a *P* values of <0.05 are shown in boldface. Raw *P* values (p.val) based on linear regression analyses are given. Age and sex were determined as important explanatory variables, and *P* values adjusted for these parameters (p.adj.age and p.adj.sex) are given as well.

10.1128/mSystems.00945-21.5 TABLE S4 Effect of precursors and dietary items on TMA-producing bacteria. Age and sex were determined as important explanatory variables, and *P* values adjusted for these parameters (p.adj.age and p.adj.sex) are given as well. Download Table S4, PDF file, 0.1 MB. Copyright © 2021 Rath et al. 2021 Rath et al. https://creativecommons.org/licenses/by/4.0/ This content is distributed under the terms of the Creative Commons Attribution 4.0 International license.

10.1128/mSystems.00945-21.6 TABLE S5 Effects of age on dietary habits, i.e., consumption of individual dietary items and contribution of overall dietary groups (rare meat consumption and daily meat consumption). Download Table S5, PDF file, 0.1 MB. Copyright © 2021 Rath et al. 2021 Rath et al. https://creativecommons.org/licenses/by/4.0/ This content is distributed under the terms of the Creative Commons Attribution 4.0 International license.

**FIG 2:**
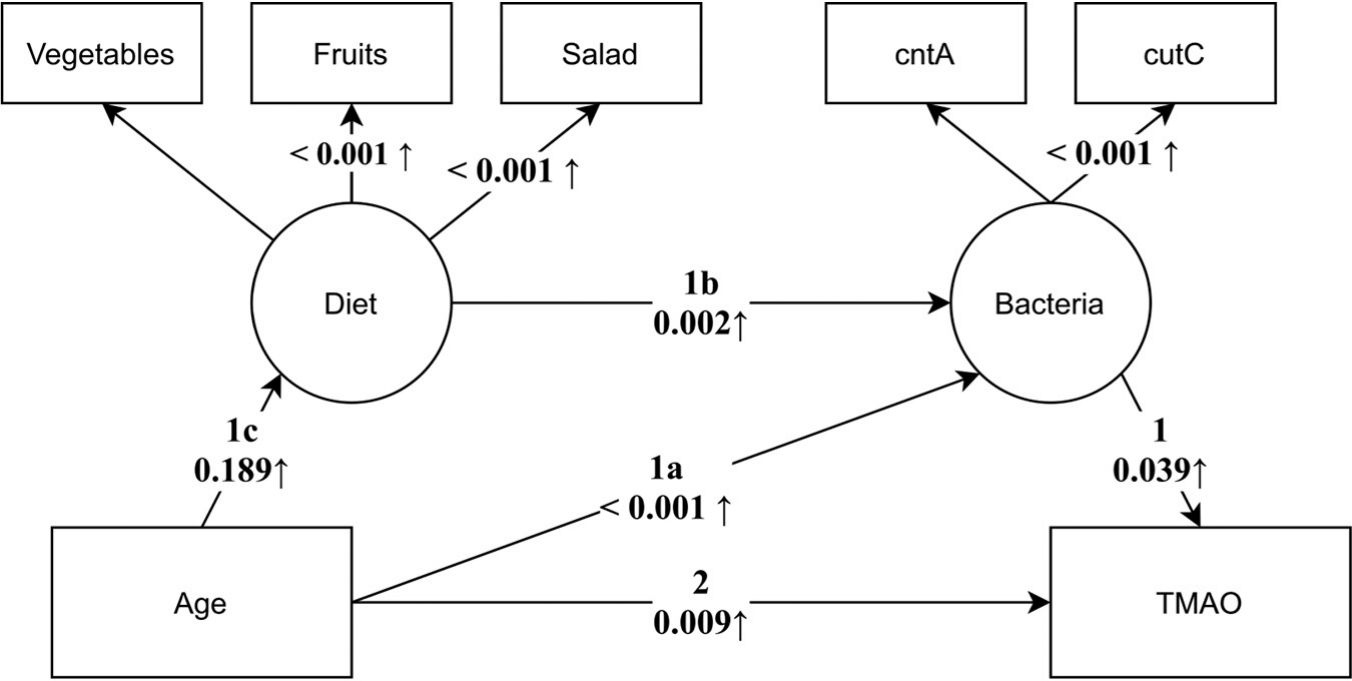
Structural equation model displaying individual routes governing increased TMAO concentrations with advancing age. Boxes refer to observed measurements, while circles represent latent constructs as measured by the measurements their arrows are pointing to. Arrows are annotated with the *P* value of the respective path coefficients. Individual routes (1a to c and 2) governing elevated TMAO levels with advancing age are shown.

Based on results of pairwise comparisons between individual components across the TMAO formation process shown in Tables 1 and 2, we constructed a structural equation model (SEM) encompassing all effects along the entire axis. Diet and potential TMA-forming communities were incorporated as latent variables defined by individual foods and gene abundances, respectively (Fig. 2). Although model construction was governed by the results of univariable analyses, the computed model fit indices indicated good fit of the model (χ^2^ test, *P* > 0.05; comparative fit index [CFI] = 0.975; root mean square error of approximation [RMSEA] = 0.033; standardized root mean square residuals [SRMR] = 0.037). The model allowed us to disentangle different routes explaining the higher TMAO levels observed with increasing age while taking into account all other routes simultaneously. The results suggest that increased abundances of potential TMA-forming bacteria (route 1a) with progressing age play a role in the process and that higher abundances of those bacteria are governed by intake of certain foods, namely, salad, fruits, and vegetables (route 1b) that are associated with age (route 1c). This effect is present on top of the direct effect of age on TMAO, which is controlled for in this model (route 2). In other words, a part of the effect of age on TMAO levels is mediated through the abundance of TMA-forming bacteria. As univariable analyses indicated effects of sex on the variables included in the SEM, we estimated an additional multiple group model and compared it to the original (restricted) model. A likelihood ratio test indicated no significant increase in model fit [χ^2^ (df = 11) = 29.53, *P* > 0.05] when sex was considered in the SEM.

As pointed out in the introduction, the role of TMAO in disease development has been demonstrated in various human cohort studies and animal models and was not the focus of this study. Nevertheless, we looked into associations between TMAO concentrations and carotid intima-media thickness (IMT) as a marker of atherosclerosis. As expected, we found a significant association between the two parameters (*P* < 0.01) that was, however, not sustained when adjusted for age (*P* = 0.192). Interestingly, stratified analyses within different age groups revealed a significant association between TMAO and IMT in only the elderly (*P* = 0.018), defined as >65 years of age, indicating that the effects of TMAO are particularly pronounced in that group (Fig. S1).

10.1128/mSystems.00945-21.1 FIG S1 Associations between carotid intima-media thickness and TMAO plasma concentration by age groups. Download FIG S1, PDF file, 0.1 MB. Copyright © 2021 Rath et al. 2021 Rath et al. https://creativecommons.org/licenses/by/4.0/ This content is distributed under the terms of the Creative Commons Attribution 4.0 International license.

## DISCUSSION

The focus of the current study was to disentangle the interplay of all major components of the TMA(O) formation process based on samples of a population-based cohort study in northeast Germany. For the first time, we were able to reveal a functional role of gut microbiota in this process. Furthermore, age was most strongly associated with plasma TMAO levels, as observed by many others in both animal experiments (24, 25) and human studies (17, 26), while other host parameters (BMI and sex) played lesser roles. Our SEM suggests that this association is partly mediated by higher abundances of TMA-forming bacteria. Moreover, the model indicated that those bacteria are promoted by foods that are low in TMA precursors but enriched in complex polysaccharides (fibers) that escape host digestion in the upper gastrointestinal tract and serve as major substrates for colonic bacteria (27). We have previously demonstrated that TMA-forming bacteria are characterized by overproportionally rich carbohydrate-active enzyme (CAZyme) repertoires that catalyze the breakdown of complex polysaccharides (22). Incongruent results have previously been reported for the effect of diet on TMAO levels. Our study suggests that overall, the abundances of the main components leading to TMAO formation, i.e., TMA precursors and TMA-forming bacteria, are not correlated but governed by distinct (dietary) factors, which might explain why an association between dietary groups and TMAO plasma concentrations was not observed here. Koay and colleagues provided another explanation for this observation, speculating that a high-fiber diet increases hepatic FMO activity, resulting in higher TMAO levels, as they observed an increased FMO gene expression in rats fed resistant starch over those on a normal chow (28). Due to its volatile nature, we were not able to quantify TMA levels, the actual product formed from gut bacteria, and hepatic FMO activity of subjects remain elusive. Along this line, it should be noted that TMA precursors were measured in plasma and do not necessarily mirror concentrations encountered by bacteria in the gut, as those compounds are also adsorbed by the host, where they are used for energy generation or metabolized to other molecules. Choline, for instance, can be oxidized to betaine or used as a precursor for acetylcholine molecules (29), and hence, both concentration and composition of TMA precursors measured in plasma probably differ from those taken up by the host through diet, independently of the TMA-forming process. Abundances of those metabolites should, hence, be considered with care in the context of this study and were not incorporated in our SEM model.

Although we uncovered important aspects of increased TMAO formation with advancing age, SEM analysis indicated that other factors not considered here might also play a role. For instance, additional pathways for TMA formation, specifically from carnitine, were recently reported (14). However, taxa and genes involved have not been elucidated so far, which hindered incorporation of this pathway in this study. Furthermore, certain archaea consume TMA via demethylation (7), and their contribution in the entire process still needs to be determined in detail. Changing host physiology and loss of function with age might be additional mechanisms leading to increased TMAO concentrations and were not determined in this study. For instance, epithelial integrity of the colon might be reduced, which is known to promote influx of bacterial metabolites, including TMAO (30). Finally, we only measured cumulative abundances of genes encoding main TMA-forming enzymes, and no information on detailed composition of their bacterial carriers, which might differ in their TMA-forming capabilities, is available. Nor did we record any data on activity, such as gene expression or protein abundances, which most probably affects TMA formation capacity of a community as well. It should be noted that expression-based parameters are, however, largely influenced by (various) environmental conditions, e.g., availability of certain food-derived substrates at a specific time point, and results are, hence, exposed to high volatility.

The age-related association of TMAO with IMT revealed in this study suggests that individuals >65 years of age are particularly affected by this hazardous metabolite. Specific age-related associations of TMAO plasma concentrations with health parameters have, to the best of our knowledge, not been reported so far in a population-based context. Experimental studies using both animal models and *in vitro* investigations on human-derived material have highlighted the contribution of TMAO to endothelial senescence and vascular aging (24, 31); however, age-specific effects of TMAO are currently unknown. Results of this study can promote experimental designs specifically assessing this issue in detail.

In conclusion, we provide crucial new information on the formation of TMAO in the general population, emphasizing the functional role of gut microbiota and specific foods in explaining increased TMAO levels with increasing age. While these results can assist the development of strategies to reduce TMAO levels, specifically in the elderly, who are prone to respective diseases, it is important that we do not propose to limit intake of foods such as salad, fruits, and vegetables that are known to largely benefit host health. Rather, our work should stimulate efforts to uncover specific factors governing TMA-forming bacteria and their subsequent therapeutic exploitations.

## MATERIALS AND METHODS

### Study participants.

Matched plasma and stool samples as well as associated metadata from 425 individuals were retrieved from the Study of Health in Pomerania (SHIP) (32, 33). Samples were specifically drawn from participants of two distinct dietary groups, namely, those consuming meat daily (*n* = 211) and those never consuming meat (*n* = 33). The latter group was expanded by samples from participants that rarely eat meat (*n* = 181), defined as “once a month or less” (*n* = 80) and “a few times per month (but less than once a week)” (*n* = 101), in order to increase sample size. Subjects suffering from chronic kidney disease and those being treated with proton pump inhibitors were excluded, as they are known to have higher TMAO levels (due to lower renal clearance) and altered gut community profiles, respectively. Genes involved in TMA formation were analyzed in all stool samples, while plasma levels of choline, betaine, carnitine, and TMAO were measured only in 383, 390, 372, and 416 samples, respectively, due to sample volume limitations in plasma samples.

### Plasma sample preparation and analysis.

Analytics were done using a label-free procedure as described previously (34). In brief, plasma samples (7.5 μl) were treated with 7.5 μl of 1% formic acid and 42.5 μl of ice-cold methanol and solutions were mixed for 10 min at 550 rpm on an Eppendorf MixMate vortex mixer. Then samples were spun down at 8°C at 3,400 rpm for 5 min and supernatants were transferred to standard high-performance liquid chromatography (HPLC) V-bottom 96-well plates (Greiner). Calibration samples containing all metabolites, namely, TMA, betaine, carnitine, and choline, at a range of 10 to 10,000 ng ml^−1^ in an artificial plasma matrix were prepared as well. All samples were analyzed using an Agilent 1290 Infinity II HPLC system coupled to an AB Sciex QTRAP 6500+ mass spectrometer. Peak areas of each sample and of the calibration curves were analyzed using MultiQuant 3.0 software (AB Sciex), and metabolites were identified based on respective ion pairs.

### Stool sample processing.

Samples were processed as previously described (8), with minor modifications. DNA was extracted from 100 to 200 mg of stool samples using the FastDNA SPIN kit for soil (MP Biomedicals, Heidelberg, Germany), including a mechanical lysis step in order to ensure DNA recovery from all bacterial taxa. Subsequently, DNA was purified with a QIAquick PCR purification kit (Qiagen, Hilden, Germany). NanoDrop analysis (260/280 and 260/230 ratios) was performed as a quality control, and DNA concentrations were quantified with the VICTOR X3 model 2030 multilabel plate reader (Perkin Elmer, Waltham, MA) using the Quant-iT PicoGreen double-stranded DNA (dsDNA) assay kit (Invitrogen, Carlsbad, CA). Only DNA samples with sufficient quality, i.e., a ratio of >1.5 both for 260/280 (expected, 1.8) and for 260/230 (expected, 2.0 to 2.2), were used for further analysis. For quantification of potential TMA-forming bacteria, the *cutC*, *cntA*, and *grdH* genes were targeted by degenerate primers and amplified in triplicate reactions in a LightCycler 480 (Roche Diagnostics) using the HOT FIREPol EvaGreen qPCR Mix Plus (no Rox; Solis BioDyne, Tartu, Estonia) and 10 ng of template DNA (8). Gene copy numbers were deduced from standard curves.

### Food frequency questionnaire and carotid artery IMT.

The food frequency questionnaire used in this study has been described elsewhere (35). Procedures for carotid ultrasound and intima-media thickness (IMT) measurements are described in reference 36. The mean thickness of the left and the right carotid arteries was used in this study.

### Statistical analyses.

Quantitative PCR (qPCR)-derived values that were below the limit of detection were replaced by the lowest observed value divided by 2 for *cutC*, *cntA*, and *grdH*. For all analyses, *cutC*, *cntA*, *grdH*, and TMAO levels were log transformed to ensure normally distributed data. Comparisons between dietary groups were performed by linear regressions with dietary status as an independent variable. To control for the effect of age, this variable was added as a covariate to the respective models. Simple linear regression models with age as a predictor were used to assess its main effects. Categorical variables were compared between groups using χ^2^ tests. All analyses were performed in the statistical programming language R (37).

The effects of microbiota on TMAO levels and of food intake on microbiota composition were assessed using linear regressions. In addition, all analyses were performed with age added as a covariate to adjust for confounding effects.

We used a structural equation model (SEM) to disentangle the complex interplay of effects. Analysis was performed using the package lavaan (38) in R. Diet and bacteria are represented as latent constructs as measured by the manifest variables fruit, vegetable, and salad intake (diet) and *cntA* and *cutC* levels (bacteria), respectively. For both latent constructs, the unstandardized coefficient of the first predictor was fixed to one to assign a scale to the latent variable. Estimation was performed using weighted least squares with robust standard errors for robustness against deviations from normality. Model fit indices in terms of the model χ^2^, comparative fit index (CFI), root mean square error of approximation (RMSEA), and standardized root mean square residuals (SRMR) were computed to indicate model fit as recommended (39). However, these indices were not used for model validation, as model construction was guided by results of previously performed univariable analyses. To assess potentially modifying confounding effects of sex, we constructed a multiple-group SEM allowing all parameters to vary between sex and compared it to the original model using a likelihood ratio test.
