# Supplementary material for: Higher Trimethylamine-N-Oxide Plasma Levels with Increasing Age Are Mediated by Diet and Trimethylamine-Forming Bacteria
Source: mSystems. 2021 Sep 14;6(5):e00945-21. doi: 10.1128/mSystems.00945-21 (PMC8547441; doi:10.1128/mSystems.00945-21)
Supplement: TABLE S1 [file msystems.00945-21-st001.pdf]

Table S1. Differences in plasma-metabolite concentrations and abundances of genes encoding key enzymes (*grdH, cntA, cutC*) of main TMA-forming pathways in stool samples of subjects by age groups. Significance (p.age) was assessed based on continuous age data.

	**18-25 (N=16)**	**26-35 (N=57)**	**36-55 (N=157)**	**56-65 (N=94)**	**66+ (N=101)**	**p.age**
**TMAO (µM)**						**<0.01**
Mean (SD)	2.23 (1.74)	2.53 (2.71)	3.18 (6.54)	3.69 (6.29)	3.60 (3.36)	
Median [Min, Max]	1.80 [0.799, 6.94]	1.84 [0.666, 20.1]	1.85 [0.479, 74.6]	2.21 [0.373, 57.4]	2.53 [0.466, 25.3]	
**Choline (µM)**						0.980
Mean (SD)	4.81 (1.16)	8.34 (3.49)	7.95 (3.51)	8.60 (3.67)	8.87 (4.28)	
Median [Min, Max]	4.46 [3.16, 7.08]	7.81 [3.54, 23.8]	7.23 [0.442, 27.7]	7.76 [0.518, 18.0]	8.03 [0.288, 22.8]	
**Betaine (µM)**						**<0.01**
Mean (SD)	13.8 (9.63)	21.7 (20.3)	18.4 (12.9)	19.8 (9.94)	22.1 (14.2)	
Median [Min, Max]	11.6 [4.13, 38.5]	15.6 [4.73, 120]	14.8 [3.32, 75.6]	18.1 [5.31, 47.4]	19.1 [1.48, 84.3]	
**Carnitine (µM)**						**0.022**
Mean (SD)	37.0 (25.1)	44.6 (22.2)	46.7 (21.8)	47.6 (20.0)	47.5 (19.7)	
Median [Min, Max]	32.4 [4.99, 112]	36.7 [15.1, 120]	44.0 [9.98, 125]	43.9 [16.5, 124]	46.5 [6.46, 101]	
***cutC* (x10^5)**						**<0.01**
Mean (SD)	1.22 (1.37)	1.09 (1.17)	1.31 (1.35)	1.67 (2.02)	1.61 (1.28)	
Median [Min, Max]	0.88 [0.05, 5.94]	0.74 [0.04, 7.22]	1.04 [0.04, 11.4]	1.21 [0.06, 15.5]	1.38 [0.09, 7.41]	
***cntA* (x10^5)**						**<0.01**
Mean (SD)	0.626 (1.59)	0.157 (0.409)	1.24 (4.99)	3.23 (15.9)	1.59 (3.91)	
Median [Min, Max]	0.04 [0.03, 6.44]	0.03 [0.03, 2.68]	0.03 [0.03, 47.5]	0.03 [0.03, 134]	0.13 [0.03, 23.4]	
***grdH* (x10^5)**						0.602
Mean (SD)	0.108 (0.096)	0.181 (0.315)	0.179 (0.355)	0.172 (0.279)	0.137 (0.203)	
Median [Min, Max]	0.07 [0.02, 0.32]	0.08 [0.01, 2.07]	0.07 [0.01, 3.24]	0.09 [0.01, 1.77]	0.07 [0.01, 1.17]	
