# Supplementary material for: Higher Trimethylamine-N-Oxide Plasma Levels with Increasing Age Are Mediated by Diet and Trimethylamine-Forming Bacteria
Source: mSystems. 2021 Sep 14;6(5):e00945-21. doi: 10.1128/mSystems.00945-21 (PMC8547441; doi:10.1128/mSystems.00945-21)
Supplement: TABLE S2 [file msystems.00945-21-st002.pdf]

Table S2. Effect of sex on TMAO, TMA precursors and TMA-producing bacteria. Diet and age were determined as important explanatory variables as well and p-values adjusted for either of these parameters (p.adj.diet; p.adj.age) are given as well.

	**Estimate**	**p.val**	**p.adj.diet**	**p.adj.age**
cutC	-0.276	**0.002**	**0.018**	**0.002**
cntA	-0.456	**0.015**	0.127	0.014
grdH	0.046	0.684	0.598	0.686
**Choline**	0.202	**0.000**	**0.002**	**0.000**
**Betaine**	0.349	**0.000**	**0.000**	**0.000**
**Carnitine**	0.200	**0.000**	**0.000**	**0.000**
**TMAO**	0.036	0.600	0.457	0.561
