# Supplementary material for: Higher Trimethylamine-N-Oxide Plasma Levels with Increasing Age Are Mediated by Diet and Trimethylamine-Forming Bacteria
Source: mSystems. 2021 Sep 14;6(5):e00945-21. doi: 10.1128/mSystems.00945-21 (PMC8547441; doi:10.1128/mSystems.00945-21)
Supplement: TABLE S3 [file msystems.00945-21-st003.pdf]

Table S3. Effect of BMI on TMAO, TMA precursors and TMA-producing bacteria. Age was determined as an important explanatory variable and p-values adjusted for this parameter (p.adj.age) are given as well.

	**Estimate**	**p.val**	**p.adj.age**
**Choline**	0.006	0.224	0.458
**Betaine**	0.003	0.596	0.627
**Carnitine**	0.011	**0.016**	0.068
grdH	0.028	**0.008**	**0.004**
cntA	-0.004	0.828	0.166
cutC	-0.002	0.787	0.184
**TMAO**	0.014	**0.032**	0.274
