# Supplementary material for: Higher Trimethylamine-N-Oxide Plasma Levels with Increasing Age Are Mediated by Diet and Trimethylamine-Forming Bacteria
Source: mSystems. 2021 Sep 14;6(5):e00945-21. doi: 10.1128/mSystems.00945-21 (PMC8547441; doi:10.1128/mSystems.00945-21)
Supplement: TABLE S4 [file msystems.00945-21-st004.pdf]

Table S4. Effect of precursors and dietary items on TMA-producing bacteria. Age and sex were determined as important explanatory variables and p-values adjusted for either of these parameters (p.adj.age; p.adj.sex) are given as well.

	Predictor	Estimate	p.val	p.adj.age	p.adj.sex
cutC	Choline	-0.106	0.218	0.127	0.482
cutC	Betaine	-0.028	0.718	0.368	0.665
cutC	Carnitine	-0.065	0.520	0.276	0.911
cutC	Fruits	0.138	**0.003**	**0.013**	**0.011**
cutC	Salad	0.036	0.248	0.152	0.523
cutC	Vegetables	0.080	**0.038**	**0.047**	0.077
cutC	Rice	-0.040	0.257	0.659	0.103
cutC	Eggs	-0.006	0.895	0.953	0.723
cutC	Full grain	-0.007	0.805	0.710	0.387
cntA	Choline	-0.271	0.143	0.070	0.300
cntA	Betaine	0.003	0.987	0.569	0.439
cntA	Carnitine	-0.265	0.224	0.097	0.522
cntA	Fruits	0.164	0.084	0.261	0.176
cntA	Salad	0.156	**0.014**	**0.005**	**0.039**
cntA	Vegetables	0.069	0.383	0.449	0.541
cntA	Rice	0.013	0.861	0.321	0.842
cntA	Eggs	-0.078	0.370	0.484	0.603
cntA	Full grain	0.098	0.083	0.103	0.198
grdH	Choline	-0.076	0.474	0.499	0.408
grdH	Betaine	-0.117	0.226	0.265	0.159
grdH	Carnitine	-0.017	0.893	0.934	0.758
grdH	Fruits	0.052	0.359	0.310	0.318
grdH	Salad	0.027	0.478	0.500	0.427
grdH	Vegetables	-0.015	0.744	0.757	0.777
grdH	Rice	0.007	0.873	0.953	0.822
grdH	Eggs	0.003	0.958	0.980	0.990
grdH	Full grain	0.059	0.083	0.080	0.065
**TMAO**	Choline	0.293	**0.000**	**0.000**	**0.000**
**TMAO**	Betaine	0.052	0.386	0.813	0.392
**TMAO**	Carnitine	0.130	0.099	0.202	0.128
**TMAO**	Fruits	0.026	0.452	0.937	0.396
**TMAO**	Salad	0.014	0.565	0.391	0.493
**TMAO**	Vegetables	0.028	0.344	0.409	0.311
**TMAO**	Rice	-0.037	0.175	0.533	0.196
**TMAO**	Eggs	0.107	**0.001**	**0.000**	**0.001**
**TMAO**	Full grain	0.023	0.274	0.320	0.225
