# Supplementary material for: Higher Trimethylamine-N-Oxide Plasma Levels with Increasing Age Are Mediated by Diet and Trimethylamine-Forming Bacteria
Source: mSystems. 2021 Sep 14;6(5):e00945-21. doi: 10.1128/mSystems.00945-21 (PMC8547441; doi:10.1128/mSystems.00945-21)
Supplement: TABLE S5 [file msystems.00945-21-st005.pdf]

Table S5. Effects of age on dietary habits, i.e., consumption of individual dietary items and contribution of overall dietary groups (Rarely Meat and Daily Meat).

	Estimate	p.val
**Fruits**	0.026	**0.000**
**Salad**	-0.005	0.349
**Vegetables**	0.006	0.335
**Rice**	-0.023	**0.000**
**Eggs**	-0.008	0.180
**Full grain**	0.011	0.050
**Rarely Meat / Daily Meat**	-0.021	**0.001**
