# Supplementary material for: Higher Trimethylamine-N-Oxide Plasma Levels with Increasing Age Are Mediated by Diet and Trimethylamine-Forming Bacteria
Source: mSystems. 2021 Sep 14;6(5):e00945-21. doi: 10.1128/mSystems.00945-21 (PMC8547441; doi:10.1128/mSystems.00945-21)
Supplement: FIG S1 [file msystems.00945-21-sf001.pdf]

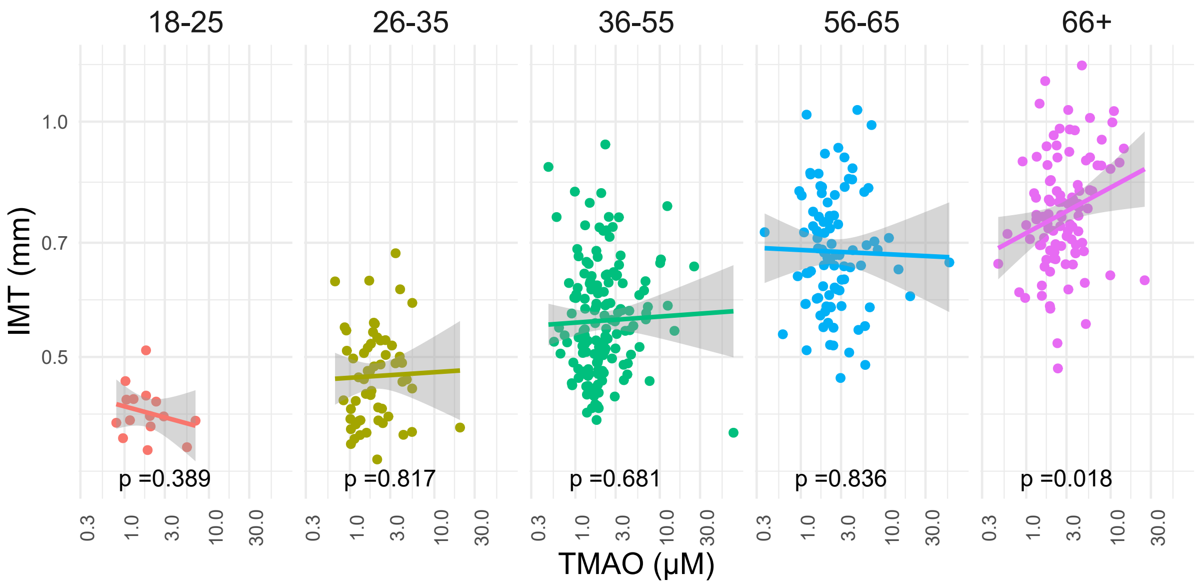

Figure S1. Associations between carotid intima–media thickness and TMAO plasma concentration by age groups.
